# Reemergence of Bolivian Hemorrhagic Fever, 2007–2008

**DOI:** 10.3201/eid1509.090017

**Published:** 2009-09

**Authors:** Patricia V. Aguilar, Wilfredo Camargo, Jorge Vargas, Carolina Guevara, Yelin Roca, Vidal Felices, Alberto Laguna-Torres, Robert Tesh, Thomas G. Ksiazek, Tadeusz J. Kochel

**Affiliations:** US Naval Medical Research Center Detachment, Lima, Peru (P.V. Aguilar, C. Guevara, V. Felices, V.A. Laguna-Torres, T. Kochel); Centro Nacional de Enfermedades Tropicales, Santa Cruz, Bolivia (J. Vargas, Y. Roca); El Servicio Departamental de Salud, Beni, Bolivia (W. Camargo); University of Texas Medical Branch, Galveston, Texas, USA (R. Tesh); Centers for Disease Control and Prevention, Atlanta, Georgia, USA (T.G. Ksiazek).

**Keywords:** Machupo virus, arenavirus, hemorrhagic diseases, Bolivia, vector-borne infections, viruses, letter

**To the Editor:** Bolivian hemorrhagic fever (BHF) was first described in 1959 during outbreaks affecting isolated human communities in eastern Bolivia. However, it was not until 1963 that the etiologic agent, Machupo virus, was isolated from the spleen of a patient who died from this disease (*1*). Although no cases were reported between 1976 and 1993, an outbreak occurred in 1994 and sporadic cases have been observed since then.

In February and March 2007, at least 20 suspected BHF cases (3 fatal) were reported to the El Servicio Departamental de Salud (SEDES) in Beni, Bolivia. In February 2007, physicians at the Hospital Santa Maria Magdalena reported 3 male patients (23, 27, and 29 years of age), who worked at a ranch in Magdalena, Itenez Province (13°14′0′′S, 64°12′0′′W). The patients sought treatment for fever, gingivorrhagia, petechiae, nausea, hematemesis, melena and tremors; clinical laboratory examinations showed thrombocytopenia (<130,000 cells/mm^3^), leukopenia (<3,900 cells/mm^3^), and hematuria. Because physicians suspected BHF, patients received supportive therapy, including intravenous hydration, corticoids, antipyretic drugs, antimicrobial drugs, and blood transfusions from donors who had survived Machupo virus infection. Nonetheless, 2 of the patients died 3 and 4 days after admission.

In February 2008, at least 200 suspected new BHF cases (12 fatal) of BHF were reported to SEDES. A febrile hemorrhagic illness developed in a 19-year-old man from Huacaraje, Itenez Province (13°33′S, 63°45′W). On first examination at the Hospital Santa Maria Magdalena, the patient had fever, tremor, gingivorrhagia, petechiae, bruises, asthenia, and anorexia and was admitted with a presumptive diagnosis of BHF. Despite supportive treatment (including administration of plasma from a BHF survivor), his condition worsened; hematemesis, melena, hematochezia, hematuria, anuria, respiratory alkalosis, and metabolic acidosis developed in the patient, eventually resulting in death. A fifth case was detected in a 46-year-old man from San Ramon, Mamore Province (13°17′0′′S, 64°43′0′′W). A febrile hemorrhagic illness developed in the patient and he was admitted to the Hospital German Busch in Trinidad. The patient recently had been hired as a farm worker. When first seen by the attending physicians, he had fever, thrombocytopenia, leukopenia, petechias, tremors, gingivorrhagia, and dehydration, consistent with symptoms of BHF. The patient received hydration, corticoids, antipyretic therapy, and a plasma transfusion from a BHF survivor. The patient’s condition improved and he was subsequently discharged from the hospital ≈10 days after admission.

Nineteen serum samples collected from suspected BHF patients, including the cases described above, were sent to Centro Nacional de Enfermedades Tropicales (Santa Cruz, Bolivia) and the US Naval Medical Research Center Detachment (Lima, Peru) for testing. Serum was injected into Vero and C6/36 cells; 10 days later, the cells were tested for flaviviruses, alphaviruses, and arenaviruses by indirect immunofluorescent assay and PCR. Five arenavirus isolates were obtained from the patients described in this report.

Viral RNA was extracted from the cell culture supernatant and the small (S) segment (≈3,200 bp) was amplified and sequenced. Phylogenetic analyses were conducted using the neighbor-joining and maximum likelihood program implemented in PAUP 4.0 software (Sinauer Associates, Inc., Sunderland, MA, USA). Sequence analyses confirmed the isolates as Machupo virus (Figure). Eight major Machupo phylogenetic lineages were described based on partial sequence of the nucleocapsid protein gene (*2*). We observed a similar tree topology based on the glycoprotein gene sequences (Figure). Two distinct lineages were distinguished among the isolates from the Itenez and Mamore provinces: V and VII and I and II, respectively. The recent isolates (2007–2008) from Magdalena and Huacaraje (Itenez Province) grouped within lineage V whereas the 2008 isolate from San Ramon (Mamore Province) belonged to lineage II. These isolates showed 10% nucleotide difference within the S segment and a 6% amino acid difference within the glycoprotein precursor gene. Similar genetic diversity has been described with Machupo virus and other arenaviruses (*2*–*4*). Sequences generated were deposited in GenBank (accession nos. FJ696411, FJ696412, FJ696413, FJ696414, and FJ696415).

**Figure:**
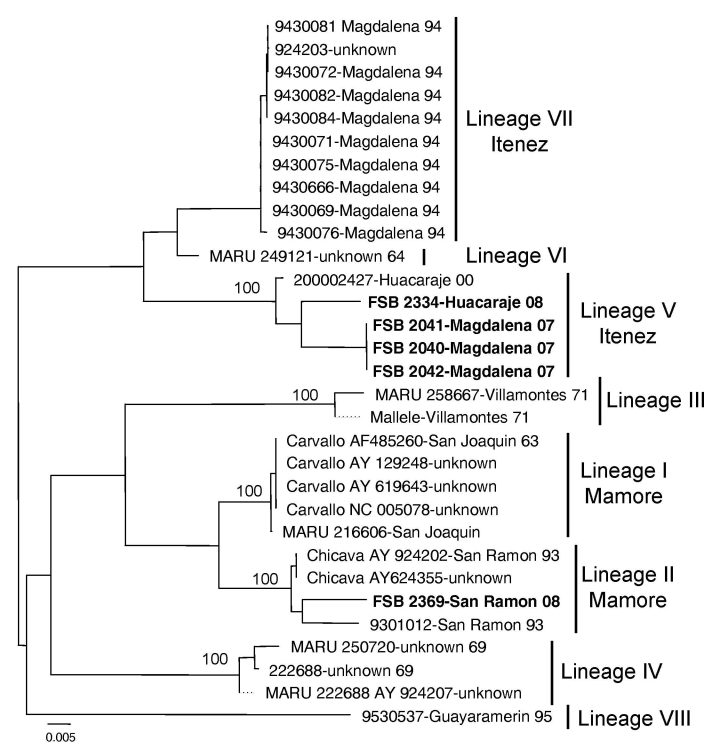
Neighbor-joining phylogenetic tree of Machupo virus derived from the glycoprotein precursor gene sequence. The neighbor-joining and maximum likelihood analyses yielded similar phylogenetic trees. **Boldface** indicates 2007–2008 isolates. Numbers indicate bootstrap values for 1,000 replicates. Scale bar indicates nucleotide substitutions per site.

It is not known whether lineage VII and I viruses continue to circulate or have been replaced by lineage V and II viruses, respectively. This study confirms the long-term maintenance of distinct phylogenetically forms of Machupo virus in a small area within Beni. Although the distribution of the Machupo virus rodent reservoir (*Calomys callosus*) extends beyond the geographic area of the Machupo cases described, factors that limit the endemic distribution of the virus remain unknown. However, population differences among *C. callosus* may account for the natural nidality of BHF (*5*). Studies are needed to fully identify and understand the ecology of the rodent reservoir and Machupo virus transmission.

Machupo virus continues to cause sporadic cases and focal outbreaks of BHF in Bolivia. We describe 5 confirmed human cases (3 fatal) of Machupo virus infection in Beni Department, Bolivia, an area in which BHF is endemic. That all 5 patients were farmers suggests their infections were probably acquired through occupational exposure. Although all the patients received plasma transfusion from patients who had survived BHF infection, 3 patients still died. An early diagnosis and the rapid administration of Machupo immune plasma before the hemorrhagic phase may increase the chance of survival, as has been observed with other arenavirus infections (*6–**8*).
